# Instruments for Measuring the Resilience of Indigenous Adolescents: An Exploratory Review

**DOI:** 10.3389/fpubh.2019.00194

**Published:** 2019-07-16

**Authors:** Crystal Jongen, Erika Langham, Roxanne Bainbridge, Janya McCalman

**Affiliations:** School of Health, Medicine and Applied Sciences, Central Queensland University, Rockhampton, QLD, Australia

**Keywords:** resilience, indigenous, adolescents, measurement instruments, socioecological resilience, cultural resilience

## Abstract

**Introduction:** Resilience is enabled by internal, individual assets as well as the resources available in a person's environment to support healthy development. For Indigenous people, these resources and assets can include those which enhance cultural resilience. Measurement instruments which capture these core resilience constructs are needed, yet there is a lack of evidence about which instruments are most appropriate and valid for use with Indigenous adolescents. The current study reviews instruments which have been used to measure the resilience of Indigenous adolescents in Canada, Australia, New Zealand, and the United States (the CANZUS nations). The aim is to provide guidance for the future use of instruments to measure resilience among Indigenous adolescents and provide recommendations for research to strengthen evidence in this area.

**Method:** Instruments were identified through a systematic search of resilience intervention and indicator studies targeting Indigenous youth from CANZUS nations. The studies were analyzed for information on the constructs of resilience measured in the instruments, their use with the targeted groups, and their psychometric properties. A second search was conducted to fill in any gaps in information. Instruments were included if they measured at least one construct of resilience reflecting individual assets, environmental resources, and/or cultural resilience.

**Results:** A total of 20 instruments were identified that measured constructs of resilience and had been administered to Indigenous adolescents in the CANZUS nations. Instruments which measured both individual assets and environmental resources (*n* = 7), or only environmental resources (*n* = 6) were most common. Several instruments (*n* = 5) also measured constructs of cultural resilience, and two instruments included items addressing all three constructs of individual assets, environmental resources, and cultural resilience. The majority of the reviewed studies tested the reliability (75%) and content or face validity (80%) of instruments with the target population.

**Conclusion:** There are several validated instruments available to appropriately measure constructs of resilience with Indigenous adolescents from CANZUS nations. Further work is needed on developing a consistent framework of resilience constructs to guide research efforts. Future instrument development and testing ought to focus on measures which include elements of all three core constructs critical to Indigenous adolescent resilience.

## Introduction

Resilience is a concept that is increasingly used to understand the factors and processes that contribute to the maintenance of well-being, effective coping, and success in the face of life's challenges (1, 2). As a strengths-based concept concerned with understanding and enhancing protective and promotive factors (1), resilience is a promising construct for researching Indigenous adolescent health and well-being. To better understand resilience for Indigenous adolescents and identify the impact of resilience-enhancing interventions, appropriate measurement instruments are needed which capture core constructs of resilience. Furthermore, these measurement instruments need to be shown to be valid, and demonstrate that they can reliably predict relevant outcomes for this population (3). In other words, measurement instruments for Indigenous adolescents need to be psychometrically sound and be shown to operationalize Indigenous concepts of well-being (4).

To be able to assess the validity and reliability of measurement instruments used to assess Indigenous adolescent resilience, we must first clearly identify the constructs of resilience which are known and often included in measurement instruments. Reaching such clarity is difficult considering there is no one definition or theoretical basis for resilience that is consistently agreed upon or used (5). This goal is also complicated by the fact that resilience is highly dependent on a person's context and culture (6, 7). Therefore, measurement instruments are required that take account of cultural diversity and/or that are tailored to specific populations and/or settings.

This review grapples with this complexity in an attempt to: increase understanding of the constructs of resilience relevant to Indigenous adolescents; appraise the inclusion of such constructs in measurement instruments which have been utilized to study Indigenous adolescent resilience; and, evaluate the psychometric properties of said instruments for use with the Indigenous adolescent populations with which they were used.

## Background

### Constructs of Resilience

Resilience has been conceptualized as a set of personality traits which assist a person to adapt positively through adversity (8). However, theorists argue that rather than a static individual characteristic, resilience can be understood as a dynamic process that is situationally and contextually embedded (5, 9, 10). Such a socioecological perspective of resilience recognizes that there are numerous protective and promotive assets and resources that serve to enhance resilience (11).

Individual assets are those intrapersonal and interpersonal skills and qualities that enable people to deal positively with emotions, work toward their desired future and maintain positive social connections. Assets include skills and qualities such as: self-efficacy; self-esteem and confidence; distress tolerance; stress management; communication skills; empathy; a balanced perspective; optimism; problem solving; goal planning and future orientation; personal awareness; and strong racial or ethnic identity (1, 12–14). Environmental resources are the support and opportunities available in a person's environment that enable positive development and successful adaptation. Such resources include: positive peer support and influence; supportive adult role models; strong family support and kinship networks; connection with members of one's cultural or social group; as well as opportunities to engage in socially valued and meaningful roles and activities (12, 14). Individual resilience-promoting assets interact with and are influenced by the resources available in a person's environment (15, 16) across multiple systems including families, peers, communities and schools (7, 12).

Concepts of resilience have existed for many Indigenous peoples even before the term resilience was coined (17). While many of the resilience-promoting individual assets and environmental resources previously outlined are recognized as universally important (18–21), there are several key cultural distinctions in the way in which Indigenous peoples conceptualize resilience (10, 22, 23). For example, family and community level factors contribute significantly more to Indigenous peoples' resilience than do individual factors (22). Cultural resilience is another key construct which may be more important for Indigenous peoples than individual-level factors (19, 22). Cultural resilience is a term that has been used to describe the degree to which the strengths of a person's culture support and promote coping (24). Cultural resilience can be strengthened through cultural connectedness, demonstrated by factors such as: a strong Indigenous identity; connections to family, community, cultural traditions, and the natural environment; and Indigenous worldviews and spirituality (17, 20, 21, 25, 26). Cultural connectedness is an important protective factor for Indigenous adolescent mental health and well-being (22, 27), and has been shown to protect against substance abuse, mental distress and suicidal behavior, and to increase prosocial outcomes such as improved socio-economic indicators and academic achievement (22, 28, 29).

### Measuring Resilience

The diversity of definitions and theories of resilience has led to the development of many measurement instruments which incorporate various combinations of individual, family, and social protective and/or risk factors (30). However, many international resilience instruments are framed within Western epistemologies and may not be valid or reliable for Indigenous people who hold differing perspectives and values (29, 31–33). Considering that constructs of resilience are culturally bound, measurement instruments are needed which are reflective of Indigenous conceptualizations and language (34, 35).

Several recent reviews of resilience measurement tools have collectively identified 19 separate instruments (30, 36, 37), many of which measure different constructs (37). Several of these instruments consider only the individual personality traits that contribute to resilience and exclude environmental resources. One review found that of four resilience scales that specifically targeted adolescents, all but one assessed only individual traits (37). Another review found that the majority of measurement instruments focused on resilience at the individual level, with only five instruments identified that adequately demonstrated a socioecological concept of resilience (30). Considering the importance of family and community level factors for Indigenous peoples resilience (22), this calls into question whether such individually focused measurement instruments would accurately measure Indigenous adolescent resilience.

Furthermore, there are examples of instruments which measure constructs of cultural resilience (25, 27); these factors are generally not measured in commonly used and recognized resilience instruments (30, 36, 37). One exception is the Child and Youth Resilience Measure (CYRM) which assesses for cultural and contextual influences on resilience (38) and has proven validity with Canadian Aboriginal youth (39) and with Indigenous Australian boarding school students (40). Its reliability and validity with other Indigenous adolescent groups is unknown.

## The Present Study

This review examines the international literature on measurement instruments that have been used with Indigenous adolescents to measure key constructs of resilience. The measurement instruments included for analysis were drawn from a systematic review of psychosocial resilience intervention and indicator studies with Indigenous adolescents in Canada, Australia, New Zealand and the United States [CANZUS nations (41)]. Specifically, we examine which constructs of resilience are reflected in the reviewed instruments and whether they capture resilience constructs that have been documented as important to Indigenous people. This review also examines the reliability and validity of the assessed measurement instruments with the Indigenous adolescent populations with whom they are used. Key issues in measuring resilience for this population group will be discussed.

## Methods

The measurement instruments reported in this exploratory review were located in publications found in a literature search conducted to identify relevant studies which aimed to either improve or measure the resilience of Indigenous adolescents in the CANZUS nations. Studies were included in this review if they reported the development, testing or utilization of instruments to measure resilience constructs with Indigenous adolescents. A separate literature review has been written by the authors on interventions to improve Indigenous adolescent resilience.

### Inclusion/Exclusion Criteria

The reviewed measurement instruments were taken from studies found in a literature search of peer-reviewed and gray literature published in English from January 1st 1990 to May 31st 2016 inclusive. The start date coincides with the third wave of resilience studies that focused on enhancing resilience by intervention (42). Publications were included if they met the following criteria:

1. The study was from Australia, Canada, New Zealand or the United States;
2. The study was focused on resilience as it pertains to Indigenous adolescents; and,
3. The study included at least one instrument which measured constructs of resilience for Indigenous adolescents.

Included publications were screened to identify measurement instruments which had been used to assess constructs of resilience with Indigenous adolescent populations. The constructs of resilience against which the measurement instruments were screened were identified through a review of the relevant literature. Key resilience publications revealed a range of resilience constructs which we grouped according to whether they reflected individual assets, environmental resources, or cultural resilience constructs for Indigenous people. See Table 1 for the constructs of resilience used to determine inclusion of measurement instruments. Measurement instruments were included for analysis if they assessed at least one construct of resilience identified in Table 1.

**Table 1:** Constructs of resilience.

**Resilience promoting factor**	**Included constructs**
Individual Assets	Self-efficacy; self-esteem and confidence; distress tolerance; stress management; problem solving, planning and decision-making skills; communication skills; empathy; personal awareness; a balanced perspective; optimism and hopefulness; future orientation; and strong racial or ethnic identity.
Environmental Resources	Supportive, positive peer relations; reliable and supportive adult role models; strong family support and kinship networks; connection with members of one's cultural or social group; positive social support networks; and opportunities to engage in socially valued and meaningful roles and activities.
Cultural Resilience	Enculturation or cultural connectedness; engagement in cultural traditions; strong Indigenous identity; connections to family, community and culture; connection to Elders; connection to the land or natural environment; and Indigenous worldview and spirituality.

### Search Strategy

**Figure 1:**
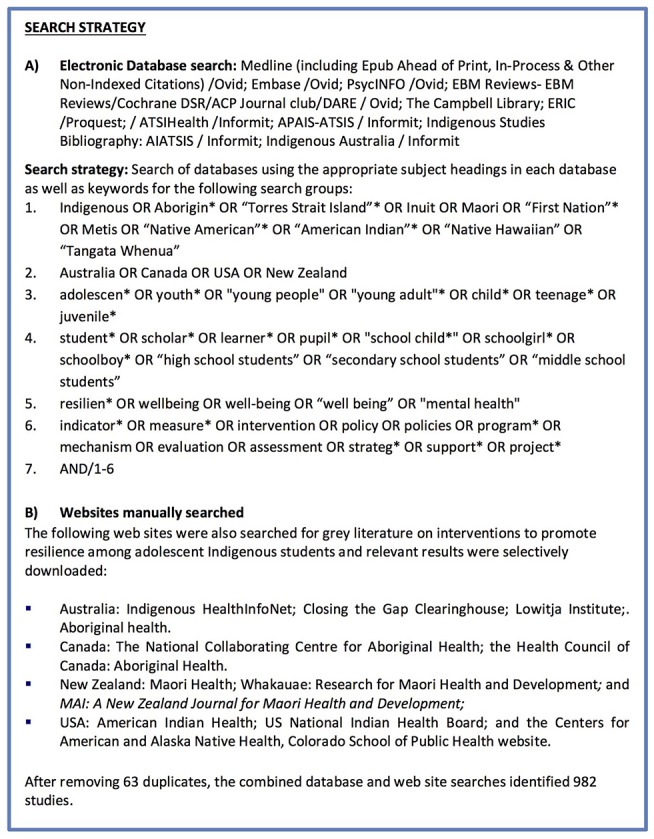
Search strategy.

The search strategy comprised five steps. See Figure 1 for summary of the search strategy.

**Figure 2:**
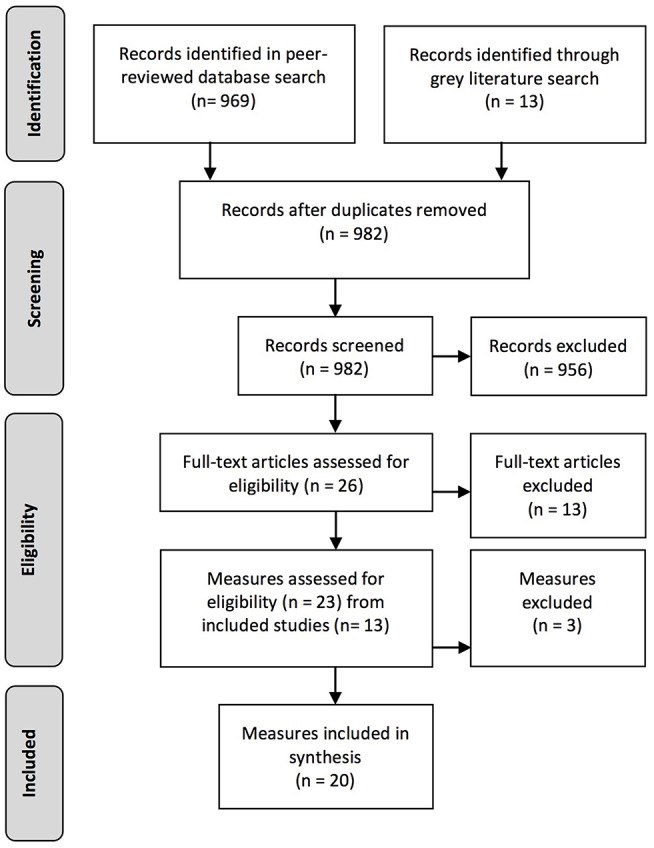
PRISMA flow chart.

**Step 1:** An expert librarian (MK) searched 11 relevant electronic databases identifying 969 references excluding duplicates.
**Step 2:** Relevant gray literature in 11 clearinghouses and websites across the four countries were searched for additional literature, locating 13 more publications.
**Step 3:** The abstracts of the 982 identified references were manually examined, with 25 studies identified for examination of full text articles. One additional relevant study known to the authors which did not appear in the search results for unknown reasons, was also included bringing the total number of studies to 26.
**Step 4:** The full-text articles of the 26 references were examined in further detail to identify the use of measurement instruments which assessed constructs of resilience. Thirteen references were excluded because they did not meet the inclusion criteria leaving 13 publications with a total of 23 measurement instruments.
**Step 5:** The included studies were examined for detail on the resilience constructs measured and instrument psychometric properties. As several of the studies from the initial search results did not include such details, a second targeted search was performed to identify further studies reporting details of specific instrument items and psychometric development and testing of the identified measurement instruments. Similar to the method employed by Ahern et al. (36), first we searched for the references on the identified measurement instruments cited in the studies included. We then conducted searches of the identified measurement instrument name, looking specifically for studies on instrument development and testing, coupled with the search terms *adolescent* and *Indigenous*. Three measurement instruments were excluded because they had either not been validated, or information on the psychometric properties of the instruments was not reported or could not be found in the second search. A total of 20 validated measurement instruments which assessed constructs of resilience for Indigenous adolescents were included for final analysis. See Figure 2 for a flow chart of our PRISMA search strategy (43).

### Identification, Screening, and Inclusion of Publications

The search results of both peer-reviewed and gray literature were imported into the bibliographic citation management software, Endnote X7 with duplicates removed. Titles and abstracts of publications were screened by one author (CJ); those which did not meet inclusion criteria were excluded. The full texts of the remaining publications were retrieved and screened by blinded reviewers (RB, JM). Inconsistencies in reviewer assessments were resolved by consensus. Included publications were screened by one author (CJ) for instruments which measure constructs of resilience.

### Data Extraction and Analysis

Data on the included measurement instruments found in the original and second search were extracted from relevant studies. For each instrument, data was extracted on the instrument type, target population and any samples utilized in studies. Data were also extracted on the explicit constructs that the included instruments measured. This was achieved by assessing individual instrument items when available, or whatever information was reported regarding the constructs measured in the relevant studies. Sometimes these very clearly reflected the resilience constructs provided in Table 1, yet often constructs were expressed using different language to that identified in the literature. Therefore, an analysis process was undertaken in which the constructs identified in instruments were compared with the core resilience constructs to identify commonalities. Details on the identified constructs measured in each instrument and the related resilience constructs can be found in the Supplementary Table 1. Each instrument was also assessed for its reported validity and reliability with Indigenous adolescents or other populations. Data on measurement validity and reliability found in the reviewed studies was extracted and is reported in detail in Supplementary Table 1.

## Results

The 13 reviewed studies included four which focused on the development and testing of resilience measurement instruments (44–47); one correlation study that examined the relationship between various instruments measuring resilience constructs (48); and eight intervention studies which utilized measurement instruments reflecting constructs of resilience for Indigenous adolescent populations (49–54), two of which were also concerned with the development and testing of appropriate measurement tools for the target populations (55, 56). Some of these studies also utilized additional instruments for measuring other mental health and well-being constructs, however these were not included in this review as they did not reflect the constructs of resilience previously specified (see Table 1). Details of the relevant studies are provided in Table 2.

**Table 2:** Included studies and their measurement instruments.

**Included Study**	**Study Outline**	**Instruments Used**
Blignault et al. (49) Intervention study	Evaluation of a community development Intervention to improve the Social and Emotional Well-being (SEWB) of Indigenous youth and improve community capacity to address youth SEWB in remote and regional areas of Australia. The GEM was used as part of the evaluation.	Growth and Empowerment Measure (GEM) ➢ Emotional Empowerment Scale (EES14) ➢ 12 Scenarios (12s)
DeJong and Hektner (50), Hall and DeJong (51), and Spears et al. (52) Intervention study	Three separate evaluation studies of a therapeutic residential model (TRM) in boarding schools for Native American children and adolescents in the US. The EQ-i was used as part of the evaluation	• BarOn Emotional Quotient Inventory (EQ-i)
Dobia et al. (56) Intervention and indicator study	Evaluation of a program to increase social connection, participation and confidence among Aboriginal girls attending secondary schools in Australia. The study aimed to determine the effects of the program on participants' resilience, connectedness, self-concept and cultural identity and sought to test culturally appropriate tools and methods for measuring these constructs.	• California Healthy Kids Survey (CHKS) ➢ Resilient Youth Development Module (RYDM) ➢ RYDM Environmental Resiliency Scale • Self-Description Questionnaire (SDQ)
Lowe et al. (53) Intervention study	Evaluation of a cultural intervention targeting substance abuse among Native American Cherokee high school students in the US. The Cherokee self-reliance scale was used to assess intervention impact	• Cherokee Self-Reliance Questionnaire (CSRQ)
Mohatt et al. (55) Intervention and indicator study	Evaluation of an intervention to increase community protective factors to support young people, reduce alcohol and other drug use/abuse, and address suicide risk among young people in Alaska Native remote communities. This study also aimed to develop unidimensional scales that are maximally sensitive to change, and described procedures used to convert longer theory-based construct mapping scales into brief measures of change.	• Elluarrluni Piyugngariluni: *Individual Characteristics (IC)* • Elluarrluteng Ilakelriit: *Family Characteristics (FC)* • Nunamta: *Community Characteristics (CC)* • Maryarta: *Peer Influences (PI)* • Umyuangcaryaraq: *Reflective Processes (RP)* • Yuuyaraqegtaar: *Reasons for Life (RL)*
Ritchie et al. (54) Intervention study	Evaluation of a culturally focused outdoor adventure intervention to promote resilience and well-being in First Nations Canadian youth. Measurement scales to examine multiple dimensions of the interconnected, holistic view of health were used, including the RS-14 and FS scales.	• 14-item Resilience Scale (RS-14) • Flourishing Scale (FS)
Snowshoe et al. (45) Indicator study	A study reporting on the development and testing of a new Cultural Connectedness scale for Canadian First Nations Métis and Inuit youth. Several positive well-being indicators were used to assess the criterion validity of the cultural connectedness scale.	• Cultural Connectedness Scale (CCS) • Measure of Adolescent Connectedness—Short Version (MAC 5-A)
Stumblingbear-Riddle and Romans (48) Correlation study	A study investigating the correlation between culture, self-esteem, subjective-well-being, and social support in fostering resilience among urban American Indian adolescents in the US.	• American Indian Enculturation Scale (AIES) • Perceived Social Support from Family (PSS-Fa) and Perceived Social Support from Friends (PSS-Fr)
Thomas et al. (47) Indicator study	A study reporting on the development and validation of a culturally appropriate tool to assess the social and emotional well-being (SEWB) of Indigenous Australian adolescents.	• Strong Souls [resilience measure] (SS)
Tomyn et al. (44) Indicator study	A study assessing the psychometric properties of a measure of subjective well-being for Indigenous Australian adolescents, and examining the correlation between subjective-well-being and general life happiness.	• The Personal Well-being Index-School Children (PWI-SC)
Williamson et al. (46) Indicator study	A study exploring the construct validity of the standard Strengths and Difficulties questionnaire (SDQ) for Aboriginal children aged 4–17 years living in urban communities in Australia.	• Strengths and Difficulties Questionnaire (SDQ) [resilience measure]

### Constructs of Resilience Measured

Of the 20 included measurement instruments, seven measured constructs of resilience consistent with the ecological definition which incorporates both individual assets and environmental resources (CHKS; SDQ-self-description; IC; FS; MAC-5A; SS; SDQ-strengths and difficulties). In contrast, there were six instruments which measured only environmental resources (FC; CC; PI; RL; PSS-Fr & PSS-Fa; PWI-SC) and three which only assessed individual assets (EQ-i; RP; RS-14). We also found three instruments which were focused on Indigenous specific constructs of cultural resilience (CCS; AIES), one of which also measured environmental resources (RL). Lastly, two identified measurement instruments addressed all three constructs of resilience, including individual assets, environmental resources, and cultural resilience (GEM; CSRQ).

The most common individual assets addressed across measurement instruments were those related to self-esteem/confidence/self-regard/self-efficacy (*n* = 8), and a future orientation, goal setting and planning (*n* = 7). Other common individual assets related to optimism and hopefulness (*n* = 4), purpose, identity and meaning (*n* = 4), problem-solving (*n* = 4), and self-reflection/self-awareness (*n* = 4). Ecological resilience constructs were very well-represented across the included instruments with connection to/support from community (*n* = 8), peers (*n* = 7), and family (*n* = 7) being common. A further four measurement instruments assessed opportunities and meaningful participation. Indigenous-specific cultural resilience constructs were included in numerous Indigenous-developed measurement instruments. Most commonly assessed was identity (*n* = 4) and Indigenous spirituality, worldviews, values and beliefs (*n* = 3). Table 3 shows the resilience constructs reflected in the reviewed measurement instruments, detailing which instruments assessed each construct.

**Table 3:** Resilience constructs measured.

**Resilience Constructs Measured**	**Measurement Instruments assessing resilience constructs**
**INDIVIDUAL ASSETS**	
Confidence/self-esteem/self-regard/self-efficacy	EQ-i; CHKS; CSRQ; IC; FS; GEM; MAC-5-A; SDQ (self-description);
Future orientation/goal setting/planning	RS-14; CHKS; CSRQ; GEM; MAC-5-A; SDQ (strengths & difficulties); RP
Purpose/meaning/identity	RS-14; FS; MAC-5-A; SS
Optimism/hopefulness	RS-14; FS; GEM; MAC-5-A
Problem solving	RS-14; EQ-I; CHKS; GEM;
Stress management/emotional coping	RS-14; EQ-i; GEM
Self-reflection/self-awareness	EQ-i; CHKS; SDQ (strengths & difficulties); RP
Empathy	EQ-i; CHKS; SDQ (strengths & difficulties);
Communication/cooperation/assertiveness	EQ-I; CHKS; GEM
Motivation/persistence	RS-14
Humor	SS
**ENVIRONMENTAL RESOURCES**	
Connection/support—Peers	CHKS; PI; MAC-5-A; PSS-Fr; SS; SDQ (self-description); SDQ (strengths & difficulties)
Connection/support—Family	CHKS; CSRQ; FC; MAC-5-A; PSS-Fa; SS; RL
Connection/support—Community	AIES; CHKS; CSRQ; GEM; CC; PWI-SC; RL
Meaningful participation/opportunities	CHKS; FS; GEM; CC
Connections/relationships general	EQ-i; FS; GEM; PWI-SC
School connection	CHKS
Role model/supportive adult	SS
Communal mastery (friends and family)	IC
**CULTURAL RESILIENCE**	
Identity	CCS; CSRQ; GEM; RL;
Spirituality/worldview/values/beliefs	CCS; CSRQ; GEM; RL
Traditions/language	CCS; CSRQ; AIES

Two of the reviewed instruments (SDQ-Strengths and Difficulties and SS) included deficit-based items assessing issues such as anxiety, depression, suicide risk, emotional symptoms, conduct problems, hyperactivity, and peer problems alongside strengths-based resilience items. Indeed, both of these instruments focused more on assessing problems, with Strong Souls (SS) including 9/25 resilience-focused items and 16/25 deficit-focus items, and SDQ including 11/25 strengths-based items and 14/25 deficit-focused items. Three other instruments reviewed (PI, RP, and RL) addressed risk factors concerning alcohol use and suicidality, yet items were framed from a strengths-based perspective, assessing resilience assets, and resources which could protect against these risks.

### Psychometric Properties of the Included Measurement Instruments

Of the 20 measurement instruments reviewed, eight (40%) were un-adapted standard instruments (EQ-I; CHKS; FS; MAC5-A; PSS-Fa & PSS-Fr; SDQ (self-description); SDQ (strengths and difficulties); PWI-SC) and five (25%) were adaptations of standard instruments (RS-14; IC; FC; PI RL). Three instruments (15%) were developed specifically for the use with Indigenous youth (CRSQ, CCS, SS), three (15%) were adaptations of instruments developed for use with Indigenous adults from the same target population (AIES; CC; RP), and one was an un-adapted instrument developed for Indigenous adults from the same target population (GEM). All eight instruments (40%) reviewed that had been adapted from their original form had been examined for reliability and some form of construct validity. Reliability, in the form of internal consistency measured by Cronbach's α, was examined for 16 (80%) of the instruments, and one study (55), which reported on six instruments also examined test-retest reliability. Excellent reliability was demonstrated for three instruments, good reliability for six, and acceptable reliability for six. However, two instruments were found to have questionable reliability, and one poor (see Table 4). Eight of the measures that reported Cronbach's α were examined with sample sizes of <100, and this included those with lower reported levels of α. Construct validity was considered in terms of both the behavior of the instrument and whether it reflects the construct being measured. Content or face validity was assessed for 16 of the instruments (80%) with community steering or advisory groups. Ten instruments (50%) demonstrated validity, as reported through examinations of convergent or discriminant validity, or some form of factor analysis. The Psychometric properties for each measurement are briefly outlined in Table 4.

**Table 4:** Instrument psychometric properties.

**Instrument/scale**	**Instrument type**	**Application/study reviewed**	**Sample**	**Reliability**	**Construct Validity**	
					**Reflective**	**Behavioral**
14 Item Resilience Scale (RS-14) (57)	Standard, one question adapted	Utilization of scale to assess effect of intervention (54)	*n* = 59 Aboriginal adolescents (Canada)	Cronbach's α = 0.78 at T1	Content validity	Not reported for this study
American Indian Enculturation Scale (AIES) (58)	Adapted version of Indigenous Developed	Application of adapted version of American Indian developed scale with American Indian adolescents (48)	*n* = 196 American Indian adolescents (USA)	Cronbach's α = 0.93	Content validity	Not reported for these studies
BarOn Emotional Quotient Inventory (EQ-i) (59)	Standard	Evaluation of therapeutic residential model (50–52)		Not reported for these studies	Not reported for these studies	Not reported for these studies
California Healthy Kids Survey (CHKS) (60)	Standard	Utilization of scale to assess effect of intervention (56)	*n* = 41 Aboriginal students and *n* = 16 non-Aboriginal adolescents (Australia)	Not reported for this study	^*^Implied content validity	^*^Implied convergent validity
Cherokee Self Reliance Questionnaire (CSRQ)	Indigenous developed	Utilization of scale to assess effect of intervention (53)	*n* = 179 Cherokee adolescents (USA)	Cronbach's *a* = 0.92	Content validity	Not reported for this study
Cultural Connectedness Scale (CCS) (45)	Indigenous developed	Development and validation of a cultural connectedness model (45)	*n* = 319 First Nations, Métis and Inuit adolescents (Canada)	Cronbach's α for each subscale Identity = 0.87 Traditions = 0.79 Spirituality −0.81	Content validity	Criterion validity Exploratory factor analysis
Elluarrluni Piyugngariluni: Individual Characteristics (IC)	Adapted version of standard measure, the Multicultural Mastery Scale (61)	Adaptation and utilization of scale to assess effect of intervention (55)	*n* = 54 Yup'ik youth (United States)	Cronbach's α Study 1 = 0.69 Study 2 = 0.79 Test-retest Study 1 = 0.80 Study 2 = 0.57	Content validity	Convergent validity Discriminant validity reported in separate publication with the same target group (61).
Elluarrluteng Ilakelriit: “Nurturing family” Family Characteristics (FC)	Standard measure the Multicultural Mastery Scale (61)	Utilization of scale to assess effect of intervention (55)	*n* = 54 Yup'ik youth (United States)	Cronbach's α Study 1 = 0.74 Study 2 = 0.72 Test -retest Study 1 = 0.48 Study 2 = 0.75	Content validity	Convergent Validity reported in separate publication with the same target group (62).
Flourishing Scale (FS) (63)	Standard	Utilization of scale to assess effect of intervention (54)	*n* = 59 Aboriginal adolescents (Canada)	Cronbach's α = 0.85 at T1	Content validity	Not reported for this study
Growth and Empowerment Measure (GEM) (64)	Indigenous developed	Utilized as an interview prompts during evaluation of youth intervention (49)	Aboriginal and Torres Strait Islander Australians and support service staff (Australia)	Not assessed in this study	Not assessed in this study	Not assessed in this study
Nunamta: “Our community” Community Characteristics (CC)	Adapted version of Indigenous Developed Protective Factors Scale (65)	Adaptation and utilization of scale to assess effect of intervention (55)	*n* = 54 Yup'ik youth (United States)	Cronbach's α Study 1 = 0.62 Study 2 = 0.52 Test -retest Study 1 = 0.62 Study 2 = 0.50	Content validity	Not reported for this study
Maryarta: “One who leads” Peer Influences (PI)	Adapted from two standard scales (66, 67)	Adaptation and utilization of scale to assess effect of intervention (55)	*n* = 54 Yup'ik youth (United States)	Cronbach's α Study 1 = 0.96 Study 2 = 0.88 Test -retest Study 1 = 0.38 Study 2 = 0.79	Content validity	Not reported for this study
Measure of Adolescent Connectedness—Short Version (MAC 5-A) (68)	Standard	Utilization of scale in validation of other scale (45)	*n* = 319 First Nations, Métis and Inuit adolescents (Canada)	Cronbach's α Present = 0.62 Future = 0.69	Implied content validity	Criterion validity (inverse)
Perceived social support from family (PSS-Fa) and perceived social support from friends (PSS-Fr) (69)	Standard	Validation with American Indian adolescents (48)	*n* = 196 American Indian adolescents (USA)	Cronbach's α = 0.89	Content validity	Not reported for this study
Self Description Questionnaire (SDQ) (70)	Standard	Utilization of scale to assess effect of intervention (56)	*n* = 41 Aboriginal students and *n* = 16 non-Aboriginal adolescents (Australia)	Not reported for this study	Not reported for this study	Not reported for this study
Strengths and Difficulties (SDQ)	Standard	Validation with Aboriginal children and adolescents (46)	*n* = 717 urban Australian Aboriginal children and adolescents (Australia)	Cronbach's α = 0.85	Content validity (71)	Convergent validity Confirmatory factor analysis
Strong Souls (47)	Indigenous developed	Development and validation of scale for Indigenous Australian adolescents (47)	*n* = 43 pilot *n* = 24 pilot *n* = 345 full study(Australia)	Cronbach's α = 0.70	Face validity Content validity	Discriminant validity Convergent validity Exploratory factor analysis
The Personal -being Index-School Children (PWI-SC) (72)	Standard	Validation with Indigenous Australian adolescents (44)	*n* = 519 Indigenous Australian adolescents (Australia)	Cronbach's α = 0.83	Not reported for this study	Principal axis factor analysis Convergent validity
Umyuangcaryaraq: “Reflecting” Reflective Processes (RP)	Adapted version of Indigenous Developed Reflective Factors Scale (73)	Adaptation and utilization of scale to assess effect of intervention (55)	*n* = 54 Yup'ik youth (United States)	Cronbach's α Study 1 = 0.49 Study 2 = 0.38 Test -retest Study 1 = 0.36 Study 2 = 0.23	Construct validity	Convergent validity Discriminant validity reported in separate publication with the same target group (74).
Yuuyaraqegtaar: “A Way to Live a Very Good, Beautiful Life” Reasons for Life (RL)	Adapted version of standard Scale (73)	Adaptation and utilization of scale to assess effect of intervention (55)	*n* = 54 Yup'ik youth (United States)	Cronbach's α Study 1 = 0.78 Study 2 = 0.69 Test -retest Study 1 = 0.71 Study 2 = 0.65	Construct validity	Not reported for this study

* *Where we have stated that validity was implied, this indicates there was a lack of sufficient details on the process of establishing validity yet some indication of validity was provided. If not otherwise stated, validity was demonstrated, meaning there was sufficient details to understand the process of determining validity*.

Supplementary Table 1 provides greater detail on the resilience constructs assessed and psychometric properties of each measurement instrument.

## Discussion

The reviewed studies reported measurement instruments to assess Indigenous adolescent resilience. The resilience constructs measured varied between studies, as did the reliability and validity of the instruments for use with the target population. We will discuss some of the key themes and issues associated with both the constructs of the instruments and their psychometric properties in turn.

### Constructs of Resilience

There is a lack of clarity and consistency in the literature regarding key definitions and concepts of resilience. This lack of clarity and consistency is reflected in resilience measurement instruments, many of which assess resilience enhancing assets pertaining to individuals, yet do not assess factors in one's environment that help to support and build resilience (30, 37). This is incongruent with much of the current resilience literature which holds that the resources and support in one's environment are critical to an individual's resilience (5, 9–11, 15, 16). There is a need for development of a clear framework on the constructs that are key to resilience to help bring consistency to this research field. Such a framework should elucidate the central role that both individual and environmental level factors play in promoting resilience. For Indigenous adolescents, such a framework would also need to incorporate cultural resilience constructs specific to the participating Indigenous peoples.

Research studies indicate that environmental resources such as connection to family and community are particularly important for Indigenous peoples, and may have a greater impact on their overall resilience than individual factors (22, 33). It was positive to see that the majority of instruments reviewed in this study included items for both individual assets and environmental resources. Although less common than measures of environmental resources, constructs of cultural resilience were also present in several instruments, particularly those developed by and for Indigenous peoples. This is important considering the central role that culture plays in Indigenous people's resilience (19, 22, 27).

Cultural resilience is understood to be about the ways in which the strengths of one's culture support and promote coping (24). However, various context dependent factors influence the ways in which people make meaning of, and take strength from culture (75). This makes measuring cultural resilience a challenge because it manifests differently across diverse Indigenous populations (19, 20), generations (76), and contexts (40). Therefore, further clarification of what cultural resilience means, and how it is expressed and experienced for different Indigenous peoples in different contexts is needed.

The constructs of cultural resilience identified in the reviewed measures included: identity; spirituality/worldviews/values/beliefs; and, traditions/language. These constructs are consistent with components of the related concept of cultural connectedness, or enculturation, which is understood to strengthen cultural resilience. The components of cultural connectedness identified in North American literature include: traditional activities; cultural identification; and traditional spirituality (77). These cultural connectedness components have been found to be valid for Canadian First Nations, Métis and Inuit youth (45), and Native American people in the US (77). However, no research examining the validity of this conceptualization of cultural connectedness with other Indigenous populations was found.

The Growth and Empowerment (GEM) instrument developed for Indigenous Australians included items related to Indigenous identity and spirituality. This suggests that Indigenous identity and spirituality may be important aspects of cultural connectedness, and therefore cultural resilience, among diverse groups of Indigenous peoples in different countries, and contexts. Nevertheless, further research into the similarities and differences in constructs of cultural resilience among different groups of Indigenous people internationally is needed to better understand this complex, context dependent concept.

Of all the reviewed instruments, only two [Growth and Empowerment (GEM) measure and the Cherokee Self Reliance Scale (CSRS)] included items assessing all three core constructs of individual assets, environmental resources, and cultural resilience constructs. Even though these are the most comprehensive measures reviewed, they were both developed for particular Indigenous contexts and may not be applicable to other populations. Further research into whether the GEM and CSRS could be appropriate for use other Indigenous adolescent populations is warranted. Additionally, future development of measurement instruments to assess Indigenous adolescent resilience should aim to ensure that instruments cover all core resilience constructs.

Interestingly, only two of the included studies incorporated instruments which have been previously identified as measures of resilience (30, 37); these were the California Healthy Kids Survey (CHKS) and the 14 item Resilience Scale (RS-14). All other included instruments measured constructs of resilience but were not identified explicitly as resilience measures. Concepts of resilience are closely related to and overlap with other constructs of well-being such as social and emotional learning and development; hence, there are a range of instruments which measure key constructs of resilience that are not explicitly identified as such. Given that many recognized resilience instruments do not measure a socio-ecological perspective of resilience and, even less so, constructs of cultural resilience, other instruments that do so may be more appropriate for measuring resilience among Indigenous adolescents.

### Instrument Reliability and Validity

One key factor limiting evidence-based practice interventions to improve well-being for Indigenous people is the lack of well-validated instruments. There is a recognized ongoing need to test and develop culturally appropriate measures to ensure they are psychometrically sound and operationalize Indigenous concepts of well-being (4). The studies revealed many different measurement instruments which have been used to study resilience with Indigenous adolescent populations across the CANZUS nations. It is promising that so many of the included measurement instruments have been tested and shown to be reliable and valid with the relevant target population. Sixteen of 20 measurement instruments assessed (80%) tested the reliability and some form of validity with the Indigenous adolescent populations with which they were being utilized.

The measurement of instrument reliability using Cronbach's α is positive, with the majority of reviewed instruments demonstrating acceptable to excellent reliability. However, Cronbach's α is not always appropriate, particularly in small samples with non-normal distributions (78, 79). Forty percent of the instruments reporting Cronbach's α were examined with sample sizes of <100 (78). This may have affected instrument reliability in some cases. To improve assessments of instrument reliability, it is important that testing is done with adequate samples.

Promisingly, content or face validity was assessed for the majority (80%) of the instruments in the studies in which they were employed. Studies' research advisory groups or steering committees' provided validation that the instruments were understandable and accurately reflected the relevant constructs in the specific cultural contexts in which they were being applied. Behavioral validity in the form of convergent or discriminant validity, or through factor analyses, was also demonstrated in 50% of instruments. The absence of reporting behavioral validity for the other instruments may be due to intervention studies included not reporting on the examination of the relationship of resilience to related constructs. Similarly, to reduce participant burden in studies of adolescents over time, additional scales of related constructs may not be included unless necessary to address the primary research question.

Other considerations in deciding on appropriate measurement instruments are the effect of age, and the test-retest reliability of instruments. Differences in comprehension levels between adolescents and children (80) and even between younger and older adolescents can differ markedly (40). Therefore, when determining the appropriateness of measurement instruments, the potential impacts of age variation should be tested. This was not examined in any of the studies reviewed. Furthermore, test-retest reliability was only examined for a handful of instruments, and several of those identified less than optimal test-retest reliability. Good test-retest reliability is important for measurement instruments intended to be used to assess changes over time and intervention outcomes. Therefore, it needs to be considered in the selection and testing of instruments for intervention studies

Due to the unique and contextualized conceptualizations of health and well-being held by Indigenous peoples, measurement instruments need to be comprehensible, and reflective of local understandings. For this reason, there is an imperative to develop measurement instruments which specifically reflect Indigenous constructs (44). Of all the instruments reviewed, 15% were developed specifically for use with Indigenous youth, and 20% for Indigenous adults, based on research on localized concepts of health and well-being, and how they can be measured. This kind of instrument development ensures a high level of content validity and cultural appropriateness which is highly important for Indigenous peoples.

However, there are also other approaches to measurement which can be equally valid. For example, in a recent study on the development of a local resilience and risk survey for Aboriginal and Torres Strait Islander young people, participants considered survey instruments developed for and/or by Indigenous Australians to be too complex or of less relevance than international measurement instruments (40). It was concluded that internationally validated instruments can be useful for measuring Indigenous adolescent well-being if they are adapted in collaboration with local communities and services and tested with local Indigenous participants (40). Considering that changes to wording or response options can result in large differences in the performance of instruments (81), the importance of testing the psychometric properties of modified or adapted instruments cannot be underestimated. In the reviewed studies, all eight adapted instruments had been examined for reliability and some form of validity.

Another consideration for the study of resilience measurement instruments for Indigenous adolescents is that localized measurement instruments, either developed or adapted for selected groups, are often not appropriate for use with the general population, and consequently data cannot be compared to that of the general population (72). An alternative is to determine the validity of mainstream instruments with Indigenous populations. This allows for the comparison of the psychometric performance of scales and health indicators or outcomes between Indigenous and non-Indigenous samples (44, 82). Two studies (44, 46) assessed the psychometric properties of mainstream instruments with Indigenous Australian children and adolescents.

This review shows that the use of tailored, adapted and standardized measurement instruments are all potentially useful approaches to measuring Indigenous adolescent resilience. However, it is important to recognize the tension that exists between having tailored measures for use with particular populations in their unique contexts, and testing standardized instruments to allow for comparability across populations.

### Measuring Resilience and Risk

Resilience, as a strengths-based construct, should arguably be measured using strengths-based instruments. However, we found some measures which assessed a greater percentage of deficit-based than strengths-based items. Determining the balance between risk related and resilience related questions when developing and choosing survey instruments can be difficult (40). Often it is important to assess for risk factors, such as suicide risk, among Indigenous adolescents, but measures of resilience also require the use of instruments which have a significant focus on strengths-based constructs. Perhaps in response to the limited availability of measures which assess an appropriate range of resilience promoting factors, several of the included studies (45, 48, 54–56), utilized multiple measurement instruments to assess various constructs related to adolescent well-being and resilience. This is one potentially feasible approach to measuring resilience among Indigenous adolescents but consideration need to be given to participant burden when administering large and complex questionnaire packages.

There are different approaches which can be taken in risk assessment. Many mainstream approaches to assessing risk may be culturally incongruent and inappropriate for use with Indigenous communities (83). To increase effectiveness, risk assessment must also be formulated in response to local cultural meanings and practices. Culturally sensitive and strengths-based approaches to assessment of risk factors was demonstrated by Mohatt et al. (55) in the Peer Influences (PI), Reflective Process (RP), and Reasons for Life (RL) instruments; PI measured protective peer influences in relation to drug and alcohol use by assessing peer attitudes that discourage alcohol or other drug use; RP assessed reflective processes about the potential consequences of alcohol use and abuse on self, family, and the Alaskan Native way of life; and RL assessed reasons why a person would not want to end their life when feeling suicidal, without mentioning suicide, emphasizing cultural beliefs and experiences that make life more enjoyable, worthwhile and meaningful. These measures were deemed by the communities involved to be more culturally sensitive than mainstream risk measures. The appropriateness and validity of such strengths-based risk assessment is something worth investigating with other Indigenous youth across the CANZUS nations.

### Limitations

Although the reviewed measurement instruments were identified through a comprehensive search strategy designed to identify the majority of peer- and non-peer reviewed literature, it is possible that some relevant publications were not found. There is a risk of publication bias considering that studies which do not find positive results are often not published. It is also recognized that many publications are not available in the most accessible international databases (84). Furthermore, as the authors are based in Australia, with longstanding skills and experience in Australian Indigenous health equity research, several known Australian Indigenous focused data bases were searched. Equivalent Indigenous health specific databases for the other included countries are not known by the authors. Additionally, other instruments known to the authors were included. As the authors are more familiar with Indigenous developed or tailored measurement instruments in the Australian context, this may have resulted in a bias toward Australian instruments.

Although terms for measures and indicators were included, because the original search was focused on resilience intervention studies, this may have also limited the results. This was not intended to be a comprehensive review. However, considering the range of instruments found which were not identified as resilience instruments, a further, more in-depth search specific to terms associated with constructs of resilience would likely reveal further instruments which measure constructs of resilience and have been utilized and validated with Indigenous adolescents.

## Summary

Core constructs of resilience for Indigenous adolescents include those relating to environmental resources and cultural resilience, as well as individual strengths and assets. To effectively measure resilience with this population group, instruments are needed which reflect and capture all core constructs. The reviewed instruments by and large reflected core resilience constructs, however only two included items reflecting all three core constructs. To assess a range of resilience constructs, many studies used multiple measurement instruments. While this is one approach to measuring Indigenous adolescent resilience, consideration needs to be given to potential participant burden. Future development, adaptation and use of instruments to measure Indigenous adolescent resilience should aim to ensure that all core resilience constructs are captured.

Instruments which assess core resilience constructs, but are not explicitly identified as resilience measures, are arguably more appropriate than known resilience measures which only capture individual resilience supporting assets. Considering the importance of environmental resources and cultural resilience in Indigenous people's resilience, measurement instruments which do not assess these may not adequately measure resilience for Indigenous adolescents. Furthermore, future studies should prioritize the assessment of cultural resilience as a core part of resilience, alongside environmental resources and individual assets. However, further research attention is needed toward understanding and clarifying specific constructs of cultural resilience in different contexts, and their similarities and differences.

The majority (75%) of the reviewed publications reported testing of instrument reliability with the target population. However, the reliability statistics of some instruments were less than acceptable, and may have been affected by small samples sizes. To gain a clearer picture of instrument reliability, it is important that testing is done with larger samples. Attention also needs to be paid to the test-retest reliability of instruments if their intended use is to measure the impacts of interventions or levels of resilience over time.

Content or face validity was assessed for the majority (80%) of the instruments in the studies in which they were employed. Research advisory groups or steering committees' provided validation that the instruments are understandable and accurately reflect the relevant constructs in the specific cultural contexts they were being applied. While using instruments developed specifically for Indigenous adolescent populations is one way to ensure strong validity and reliability, this may not always be the best approach. The adaptation of instruments designed for other populations is another valid approach if shown to be psychometrically sound. Also, the benefits of testing the reliability and validity of non-adapted mainstream instruments with Indigenous people, is that if shown to be reliable and valid they can facilitate the comparison of intervention effects with those of their mainstream counterparts.

## Conclusion

This review demonstrates that there is a range of instruments which have been successfully used to measure resilience with Indigenous adolescent populations in CANZUS nations. While the majority of these instruments are not well-established and commonly used resilience measures, they strongly reflected the core constructs of resilience for Indigenous people. The majority of reviewed instruments were also shown to be reliable and valid for use with the target populations. When choosing instruments to measure Indigenous adolescent resilience, key considerations are the selection of instruments which reflect core resilience constructs and the testing of instrument reliability and validity. This review provides examples of potential instruments which could be used in future studies, as well as guidance for the testing and development of further instruments to measure Indigenous adolescent resilience.

## Author Contributions

RB and JM designed the study protocol used for the search. CJ was responsible for the initial screening of search results and data extraction for included studies and RB and JM both completed second screening. CJ was primarily responsible for writing the review draft, and RB and EL contributed to the drafting of the review. CJ, RB, JM, and EL all contributed feedback and edited the review. All authors have approved the final version for submission and agree to be accountable for all aspects of this work.

### Conflict of Interest Statement

The authors declare that the research was conducted in the absence of any commercial or financial relationships that could be construed as a potential conflict of interest.

## Abbreviations

AIES: American Indian Enculturation Scale
CANZUS: Canada, Australia, New Zealand, United States
CC: Nunamta: *Community Characteristics*
CCS: Cultural Connectedness Scale
CHKS: California Healthy Kids Survey
CRSQ: Cherokee Resilience Scale Questionnaire
CYRM: Child and Youth Resilience Measure
EQ-i: BarOn Emotional Quotient Inventory
FC: Elluarrluteng Ilakelriit: *Family Characteristics*
FS: Flourishing Scale
GEM: Growth and Empowerment Measure
IC: Elluarrluni Piyugngariluni: *Individual Characteristics*
MAC 5-A: Measure of Adolescent Connectedness – Short Version
PI: Maryarta: *Peer Influences*
PRISMA: Preferred Reporting Items for Systematic Reviews and Meta-Analyses
PSS-Fa: Perceived Social Support from Family
PSS-Fr: Perceived Social Support from Friends
PWI-SC: The Personal Well-being Index-School Children
RL: Yuuyaraqegtaar: *Reasons for Life*
RP: Umyuangcaryaraq: *Reflective Processes*
RS-14−14-item: Resilience Scale
RYDM: Resilient Youth Development Module
SS: Strong Souls
SDQ: Self-Description Questionnaire
SDQ: Strengths and Difficulties Questionnaire.
