# Supplementary material for: Instruments for Measuring the Resilience of Indigenous Adolescents: An Exploratory Review
Source: Front Public Health. 2019 Jul 16;7:194. doi: 10.3389/fpubh.2019.00194 (PMC6647871; doi:10.3389/fpubh.2019.00194)
Supplement: Supplementary file 1 [file Table_1.docx]

Supplementary Table 1: Instrument Psychometric Properties

| **Instrument, instrument type, sample, and target population** | **Identified Constructs**  **Measured** | **Related Resilience**  **Constructs** | **Reliability** | **Validity** |
| --- | --- | --- | --- | --- |
| **14-item Resilience Scale (RS-14)**  A standard measurement instrument which operationalizes trait-like conception of resilience focused on personal characteristics [1]. A one question adapted version of the instrument was used with a sample of *n=*59 Aboriginal Canadian Youth. The adapted version was found to have acceptable reliability and content validity for use with the target population in the included study [2]. | **14-item Resilience Scale (RS-14)**   - Measures five core characteristics of resilience including: meaning and purposeful life; perseverance; equanimity; self-reliance; and existential aloneness [1]. | **14-item Resilience Scale (RS-14)**   - Measures individual assets of: meaning and purpose; goals and values; perseverance, motivation and persistence; ability to deal with stress; optimism and future orientation; problem solving skills; and independence [1]. | Demonstrated internal consistency of Cronbach’s *a* = .78 with study participants [2]. | *Content validity –* The project was conducted by a collaborative research team comprising health leaders from Wikwemikong Unceded Indian Reserve and academic researchers. A local Community Research Steering Committee (CRSC) in Wikwemikong provided oversight and guidance through all phases of the research and the development of the survey package, including the review and selection of all scales and questions included. The CRSC included two adolescent representatives. the CRSC evaluated and selected the most appropriate scales that were to be included, and the final version of the HWBQ package of scales was approved for use in the community [2]. |
| **American Indian Enculturation Scale (AIES)**  A measurement instrument of American Indian (AI) enculturation developed for Native American adults [3], adapted for use with American Indian adolescents [4]. The instrument was found to have excellent reliability and content validity with a sample of *n=*196 American Indian adolescents in the USA included in the study [4]. | **American Indian Enculturation Scale (AIES)**   - Measures the level of enculturation among AIs by determining their participation in traditional behaviors and practices [4] | **American Indian Enculturation Scale (AIES)**   - Measures cultural resilience constructs for Indigenous people regarding the importance of connecting with culture and community. | Demonstrated excellent reliability with internal consistency of Cronbach’s *a* = .93 with American Indian adolescent study participants [4]. | *Content validity –* The AIES was developed for American Indian adults through expert opinion, review of the literature, and feedback from diverse American Indian people as cultural experts [3]. In the study, a community advisory board composed of AI professionals and community members provided feedback on questionnaire development and gave approval for the use of the adapted version of the scale the study [4].  ***Convergent and discriminant validity* was demonstrated in a sample with American Indian adults**. Pearson correlational analyses *AIES* was significantly and positively correlated with the behavioral (*p* < .01) and affective/spiritual (*p* < .01) acculturation subscales of the *LPS*, indicating convergent validity. Further, the *AIES* correlated negatively with the *NAAS* (*r* = -.68, *p* <.01), indicating discriminant validity from acculturation [3]. |
| **BarOn Emotional Quotient Inventory (EQ-i)**  A standard measurement instrument of emotional and social intelligence (ESI) originally designed for adults [5]. A youth version has been tested and shown to be valid with Canadian Aboriginal youth in a separate study [6], however the included studies did not state which version of the instrument was used and **validity and reliability with the study populations was not reported** [7-9]. | **BarOn Emotional Quotient Inventory**  **(EQ-i)**   - Self-report measure of emotionally and socially intelligent behaviour including 5 composite scales: intrapersonal; interpersonal; stress management; adaptability; and general mood. | **BarOn Emotional Quotient Inventory (EQ-i)**   - Measures internal assets including:   self-regard; emotional self- awareness; assertiveness; empathy; interpersonal relationship; stress management; adaptability; problem-solving; and optimism. |  | Results of a **Confirmatory factors analysis (CFA) of the youth form** for the EQ-i (EQi:YV) provide empirical support for the **validity of the EQ-i:YV’s four-factor structure with Canadian aboriginal children and adolescents** [6]. |
| **California Healthy Kids Survey (CHKS**)  A standard youth measurement instrument, the CHKS Resilient Youth Development Module (RYDM) and RYDM Environmental Resiliency Scale assess internal assets and environmental resources associated with positive youth development and school success [10]. The instrument has implied content and convergent validity with a sample of  *n*=41 Australian Aboriginal adolescent students. However it’s **reliability and other forms of validity** with study population is **unknown** [11]. | **Resilient Youth Development Module (RYDM)**   - Includes subscales assessing personal social-emotional skills including: self-efficacy; empathy; problem solving; self-awareness; cooperation and communication; and goals and aspirations.   **RYDM Environmental Resiliency Scale**   - Includes subscales assessing dimensions of relatedness including: school connectedness; school support; school meaningful participation; community support; community meaningful participation; peer caring relationships; prosocial peers; home meaningful participation; and home support. | **Resilient Youth Development Module (RYDM)**   - Each subscale directly measures core resilience constructs of internal assets in the form of intrapersonal and interpersonal skills.   **RYDM Environmental Resiliency Scale**   - Each subscale directly measures core resilience constructs of environmental resources through connection and support with peers, family, school and community as well as meaningful participation. | Assessed **with a sample of 12,000 ethnically diverse high school students in the USA** from grades 7, 9 and 11. The secondary school RYDM scales demonstrate acceptable levels of reliability, with all scales exhibiting reliabilities greater than 0.70, and 11 of 13 scales demonstrating reliabilities greater than 0.75. The school support, community support, and peer caring relationships scales exhibit the highest internal consistency, with alphas all exceeding 0.90. Internal consistency does not differ markedly by student grade, gender, or race/ethnicity. The secondary school RYDM scales exhibit low test-retest reliability, with 8 of the 12 scales exhibiting pre-post correlations of less than 0.60. This suggests that the module is not well suited for examining student-level changes over time [10]. | *Implied Content validity –* Community consultation with community Elders and organisations as well as Aboriginal Girls Circle leaders, program developers and school-based personnel informed the selection and adaptation of the CHKS [11] implying content validity. However further details of this process were not provided.  *Convergent validity:* Was implied through the assessment of correlations between internal resilience factors and mental health, self-esteem and total environmental resiliency [11].  *Construct validity -* the secondary school RYDM instrument provides a valid assessment of environmental resilience assets **with a sample of 12,000 ethnically diverse high school students in the USA** because these constructs are associated with student substance use, depression, self-reported grades, truancy, and test scores in expected ways. However, two of the six internal assets — cooperation and goals/aspirations—could not be assessed validly [10]. |
| **Cherokee Self-Reliance Questionnaire (CSRQ)**  Indigenous developed measurement instrument to reflect local constructs of wellbeing and connectedness, developed for Cherokee adults [12]. The instrument was found to have excellent reliability and content validity with a sample of *n*=179 Cherokee adolescentsin the USA [13]. | **Cherokee Self-Reliance Questionnaire (CSRQ)**   - Cherokee self-reliance is a composite of three categories including (a) being responsible *(Caring for Self* & *Others***)**, (b) being disciplined *(Setting* & *Pursuing Goals)*, and (c) being confident *(Having a Sense of Identity* & *Self Worth)*. Two cultural themes cut across all three categories: (a) being true to oneself and (b) being connected. | **Cherokee Self-Reliance Questionnaire (CSRQ)**   - Measures internal assets such as self-worth or self-esteem, future orientated goal setting and persistence, environmental assets of connection to family, community, and tribe, and constructs of cultural resilience including strong Indigenous identity, connection to worldview, values and beliefs, and honoring traditional ceremonies and the Cherokee language. | The study reported a Cronbach’s *a* = .92 [13]. | *Content validity* – **A** Community Partnership Steering Committee (CPSC) of 6 Keetoowah-Cherokee community representatives led by a Keetoowah-Cherokee tribal elder who served as the Community Liaison/Interventionist selected and reviewed the most culturally appropriate measures for substance abuse and stress. The instruments were pilot tested with Keetoowah-Cherokee adolescents. The adolescents provided recommendations and feedback [13].  This tool was **developed for Cherokee adults** based on Cherokee cultural concepts of self-reliance through ethnographic research with Cherokee adults. The questionnaire relates to Cherokee way of life and is composite of three major components of Cherokee cultural values and beliefs [12]. |
| **Cultural Connectedness Scale (CCS)**  A measurement instrument of cultural connectedness developed for First Nations Canadian youth. The instrument was found to have acceptable to good reliability for all sub-scales, content validity and criterion validity with a sample of *n*=319 First Nations, Métis and Inuit adolescents in Canada [14]. | **Cultural Connectedness Scale (CCS)**   - Includes three factors: identity (positive sense of exploration and commitment to one’s culture), traditions (utility of traditional practices and language), and spirituality (connection to the spirit world through an adoption of a FN worldview). | **Cultural Connectedness Scale (CCS)**   - Measure cultural resilience constructs specific to Indigenous people related to the importance of a strong, positive identity, and connection to culture through practicing traditions and language, and connection to a deeper Indigenous worldview. | Tested with sample of 319 FN, Métis, and Inuit youths enrolled in Grades 8–12 from reserve and urban areas in Canada. Confirmatory factor analysis resulted in a 29-item inventory consisted of 3 dimensions: identity, traditions, and spirituality [14].  All three subscales demonstrated adequate scale score reliabilities (Cronbach’s alpha .872 for identity, .791 for traditions, and .808 for spirituality). The Pearson’s *r* correlations among the three scales ranged from .49 to .69, indicating that these measures represent an underlying common construct of cultural connectedness as well as unique aspects associated with identity, traditions, and spirituality. | *Content validity –* A two-stage process (development and judgment-quantification) was used for rigorous instrument development. Instrument development included domain identification (informed by cultural experts and FN, Métis, and/or Inuit youth), and measure formation (local school board’s FN, Métis, and Inuit student achievement committee evaluated the face validity, relevance, clarity and unambiguousness of the items and made appropriate revisions). Judgment-quantification was achieved using a content validity index (CVI) with expert judges selected based on their extensive knowledge in cultural connectedness for FN youth. The item selection stage was based on inspection of rational expert judgments (i.e., CVIs) and the participant responses to the items (i.e., participant endorsement frequencies) [14].  *Criterion validity –* The Satisfaction with Life Scale for Children (SWLS–C), Heming- way Measure of Adolescent Connectedness—Short Version (MAC 5-A), and spiritual attendance items were used as positive well-being indicators to assess criterion validity of the cultural connectedness scales. All correlations between the cultural connectedness scales and their theoretically relevant measures were significant and in the expected direction, providing evidence for criterion validity [14]. |
| **Elluarrluni Piyugngariluni: ‘‘Learning in the Mind of Doing Things in a Masterful Way’’ *Individual Characteristics (IC)***  A measurement instrument of sense of efficacy based on the Communal Mastery: Family and Communal Mastery: Friends subscales from the Multicultural Mastery Scale (MMS) for youth [15] adapted for use with Alaska Native youth. The instrument was found to have acceptable internal reliability and test-retest reliability in one study with *n*=54 Yup’ik Alaska Native youth. Content validity was demonstrated in the study, and convergent and discriminant validity of the instrument was demonstrated in another study with the same target group by the authors [16]. | **Individual Characteristics (IC)**   - Consists of three subscales measuring sense of mastery through joining with friends (Mastery-Friends), through joining with family (Mastery-Family), and through one’s own efforts (Mastery-Self). | **Individual Characteristics (IC)**   - Measures internal assets of self-efficacy, problem solving, coping and stress management and environmental resources through communal mastery – one’s belief in capacity to solve life’s challenges and have control over goal achievement with friends and family. | The study reported a Cronbach’s *a* = .69 in study 1 and Cronbach’s *a* = .79 in study 2. Test–retest Reliability for Study 1 = .80, and study 2 =.57 [16]. | *Content validity –* Content validity was established through expert review, cultural expert consultation, pilot testing, and further review to ensure equivalence with core concepts/original measures and cultural appropriateness [16].  **Convergent and discriminant validity were shown in a separate study on instrument adaptation with Alaska Native adolescents.** *Convergent validity –* The MMS scores correlated positively with the Reasons for Life (RFL) scores (*r* .51, *p* .01) and the Youth Community Support scores (*r* .41, *p* .01) [15]. *Discriminant validity* – The absence of a significant association between MMS scale scores and Religious Involvement scores (*r* .06, *p* .28) provided evidence for discriminant validity [15]. |
| **Elluarrluteng Ilakelriit: ‘‘Nurturing family’’ *Family Characteristics (FC)***  19-item version of the Brief Family Relationship Scale (BFRS), a measurement instrument of family connectedness and relationships adapted from the relationship dimension of the mainstream Family Environment Scale for use with Alaska Native youth [17].  The instrument was found to have acceptable internal reliability in both studies and acceptable test-retest reliability in one study with *n*=54 Yup’ik Alaska Native youth. Content validity was demonstrated in the study, and convergent and discriminant validity of the instrument was demonstrated in another study with the same target group by the authors [16]. | **Family Characteristics (FC)**   - Consists of three subscales assessing youth perceived family cohesion, expressivity, and conflict. | **Family Characteristics (FC)**   - Measures environmental resources of family togetherness/relatedness, and support. | The study reported a Cronbach’s *a* = .74 in study 1 and Cronbach’s *a* = .72 in study 2. Test–retest Reliability for Study 1 = .48, and study 2 = .75 [16].  **In a separate study on instrument adaptation with Alaska Native adolescents** internal consistency was acceptable for Cohesion (α = .83) and Conflict (α = .80) and for the full-scale BFRS (α = .88) but weaker for Expressiveness (α = .65) indicating a potentially poor fit of the construct of expressiveness in this non-Western cultural group [17]. | *Content validity –* Content validity was established through expert review, cultural expert consultation, pilot testing, and further review to ensure equivalence with core concepts/original measures and cultural appropriateness [16].  **Convergent validity was shown in a separate study on instrument adaptation with Alaska Native adolescents** *–* The BRFS scores correlated in the expected direction with the *Communal Mastery Family Scale (*α = .76) scores, the *Reasons for Life Scale (*α = .83) scores, and the *Youth Community Protective Factors Scale (*α = .77) scores [17]. |
| **Flourishing Scale (FS)**  A standard measurement instrument of subjective-wellbeing designed to measure social-psychological prosperity developed for use with adults [18]. The instrument was found to have good reliability and content validity for use with *n*=59 Canadian Aboriginal adolescents [2]. | **Flourishing Scale (FS)**   - Measures respondents self-perceived success in areas of relationships, self-esteem, purpose and optimism. | **Flourishing Scale (FS)**   - Measures individual assets (including competence and capability, purpose, meaning, self-acceptance and optimism) and environmental assets (including supportive relationships, and engagement and interest in activities which have meaning and purpose). | Demonstrated internal consistency of Cronbach’s *a* = .85 at Time 1 with study participants [2]. | *Content validity –* The scale was reviewed and approved by a local community research steering committee which included two adolescent representatives [2]. |
| **Growth and Empowerment Measure (GEM)**   - Emotional Empowerment Scale (EES14) - 12 Scenarios (12s)   The Growth and Empowerment Measure (GEM) was an instrument developed to measure change in dimensions of empowerment as defined and described by Aboriginal Australians adults who participated in the Family Well Being program (FWB). It’s reliability and validity were assessed with a sample of adults in Australia identifying as Aboriginal and/or Torres Strait Islander (median age 40). Included participants from urban, regional and remote areas [19]. The **reliability and validity** **with target population** of Aboriginal adolescents is **unknown.** | **Emotional Empowerment Scale (EES14)**   - Consists of two subscales: inner peace and self-capacity.   **12 Scenarios (12s)**   - Consists of two subscales: healing and growth; and connection and purpose. | **Emotional Empowerment Scale (EES14)**   - Measures internal assets including confidence, stress management, problem solving, self-efficacy, communication skills, hopefulness, and environmental resources of connection and opportunity.   **12 Scenarios (12s)**   - Measures of internal assets including dealing with difficult emotions, future orientation, communication skills, environmental resources of relationships and community connectedness, and cultural resilience constructs of sense of identity and spirituality. | **With a sample of Aboriginal and/or Torres Strait Islander adults [19]:**  The EES14 scale showed high internal consistency (Cronbach’s Alpha 0.891). Individual items had minimal impact on the scale mean, variance or alpha, and all corrected item-total correlations exceeded 0.40.  Empowerment scenario responses showed strong internal consistency with a Cronbach’s alpha of 0.856. Deletion of individual items had minimal impact on the scale mean, variance or alpha. | *Content validity* – developed through work with Aboriginal FWB program participants and expert consultation and piloting with FWB participants to reflect Aboriginal constructs of social and emotional wellbeing [19].  *Construct validity* – Construct validity was assessed through correlation with other established wellbeing measures: Kessler Distress Scale (K6) and two questions regarding general mood. Most scores from the three measures inter-correlated strongly [19]. |
| **Nunamta: ‘‘Our community’’**  ***Community Characteristics (CC)***  The Community Characteristics (CC) measurement instrument [16] is based on the Youth Community Protective Factors Scale which was adapted from the Yup’ik Protective Factors scale developed for adults describing elements of protective communities [20]. Content validity was demonstrated with *n*=54 Yup’ik Alaska Native youth, however reliability in this sample was suboptimal [16]. | **Community Characteristics (CC)**   - Assesses youth perceived community protective factors. The items comprise two subscales tapping Support (3 items) and Opportunity (4 items). | **Community Characteristics (CC)**   - Measures environmental resources of community support and opportunities. | The study reported a Cronbach’s *a* = .62 in study 1 and Cronbach’s *a* = .52 in study 2. Test–retest Reliability for Study 1 = .62, and study 2 = .50 [16].  In a **separate study with Alaska Native youth**, community characteristics subscales displayed acceptable internal consistency for support (.76) and questionable internal consistency for opportunities (.60) [20]. | *Content validity –* Content validity was established through expert review, cultural expert consultation, pilot testing, and further review to ensure equivalence with core concepts/original measures and cultural appropriateness [16]. |
| **Maryarta: ‘‘One who leads’’**  ***Peer Influences (PI)***  The 10-item Peer Influences (PI) measurement instrument [16] was adapted from two scales from the American Drug and Alcohol Survey: The Peer Discouragement of Alcohol, Tobacco, and Other Drug (ATOD) Use Scale (Discourage) and the Disapproval of Peers’ ATOD Use Scale (Disapproval) [20]. The instrument was found to have good to excellent internal-consistency reliability, and acceptable test-retest reliability in one study as well as demonstrated content validity *n*=54 Yup’ik Alaska Native youth [16]. | **Peer Influences (PI)**   - Assesses peer attitudes that discourage alcohol or other drug use. | **Peer Influences (PI)**   - Measures environmental resources of protective peer influences in relation to drug and alcohol use. | The study reported a Cronbach’s *a* = .96 in study 1 and Cronbach’s *a* = .88 in study 2. Test–retest Reliability for Study 1 = .38, and study 2 = .79 [16].  In a **separate study with Alaska Native youth**, peer influences subscales demonstrated good internal consistency with alpha coefficients: discourage (.86) and disapproval (.88) [20]. | *Content validity –* Content validity was established through expert review, cultural expert consultation, pilot testing, and further review to ensure equivalence with core concepts/original measures and cultural appropriateness [16]. |
| **Measure of Adolescent Connectedness – Short Version (MAC 5-A)**  A standard measurement instrument of adolescent connectedness and belonging over time [21]. The instrument subscales had questionable reliability, and content and inverse convergent validity were implied with a sample of *n*=319 First Nations, Métis and Inuit adolescents in Canada [14]. | **Measure of Adolescent Connectedness (MAC 5-A)**   - Includes a self in present subscale assessing positive connections in youth’s lives, including connection to family, friends, school and self; and, a self-in future subscale assessing positive qualities of youth that are perceived by other and action taken by youth to secure a positive future. | **Measure of Adolescent Connectedness (MAC 5-A)**   - Measures individual assets of self-esteem, identity, goal setting and hopefulness for the future, and environmental resources of positive connection and support with peers and family. | The study reported a Cronbach’s *a* = .62 for the sense of self in the present subscale, and Cronbach’s *a* = .69 in the sense of self in the future subscale [14].  **Other studies** have found adequate internal consistency coefficients for MAC 5-A Scales for **both genders and across diverse ethnic groups in an adolescent sample**, indicating good reliability. Test-retest and inter-item reliability estimates were satisfactory. All but two of the scales demonstrated estimates of reliability in the good (.70 - .80) to very good (.80 - 90) range [21]. | *Implied Content Validity:* The MAC 5-A was assessed in suitability to establish validity of the Cultural Connectedness Scale (CCS) being developed by the authors, indicating implied content validity,  *Criterion validity (inverse):* The MAC 5-A was used as a positive wellbeing measure to assess the criterion validity of the CCS, therefore showing implied criterion validity.  **In a normative adolescent sample:** *Construct validity –* Correlations between other measures (including the school & Family Connectedness Scales (family; α = .78 & school; α = .72), Reason for Living Inventory for Adolescents (α = .83), Social Connectedness Scale II (α = .93) and the inventory of Parent and Peer Attachment), and the connectedness subscales and composite scales provided evidence of construct validity [21]. |
| **Perceived Social Support from Family (PSS-Fa) and Perceived Social Support from Friends (PSS-Fr)**  A standard measurement instrument of social support defined as the extent to which an individual believes their needs for support, information, and feedback are fulfilled [22]. The instrument was found to have good reliability and content validity with a sample of *n*=196 American Indian adolescents in the USA [4]. | **Perceived Social Support from Family (PSS-Fa) and Perceived Social Support from Friends (PSS-Fr)**   - A measure used to determine self-perceived levels of social support from family and friends. | **Perceived Social Support from Family (PSS-Fa) and Perceived Social Support from Friends (PSS-Fr)**   - Measures environmental resources in the form of support from and connection to peers and family. | In the current study, with American Indian adolescents the Cronbach’s alpha coefficients for social support from family and social support from friends scales was .89 [4]. | *Content validity -* A community advisory board composed of AI professionals and community members provided feedback on questionnaire development and selection [4]. |
| **Self-Description Questionnaire (SDQ)**  A standard measurement instrument of adolescent self-concept [23]. **Reliability and validity with the target population unknown.** | **Self-Description Questionnaire (SDQ)**   - Includes subscales assessing forms of adolescent self-concept and general wellbeing including: general self-esteem; parental self-concept; enjoyment; musical self-concept; art self-concept; academic self-concept; emotional stability; physical appearance. | **Self-Description Questionnaire (SDQ)**   - Measures internal assets, such as self-esteem and general skills and abilities, as well as environmental resources through relationship with parents. | Within a **normative sample of high school adolescents** reliabilities for the 11 SDQII-S factors were nearly the same and consistently high (.80 to .89) for two groups [23] | Within a **normative sample of high school adolescents** multiple-group confirmatory factor analyses revealed that the factor structure based on responses to 51 items by a new cross-validation group (*n* 9,134) was invariant with the factor structures based on responses to the same 51 items and to all 102 items by the original normative archive group (*n* 9,187). Multi trait–multimethod analyses support the internal validity of responses over time [23]. |
| **Strengths and Difficulties Questionnaire (SDQ)**  A standard measurement instrument measuring children strengths and difficulties over the past 6 months. The instrument was found to have good reliability, as well as content and convergent validity with a sampel of *n*=717 urban Australian Aboriginal children and adolescents [24]. | **Strengths and Difficulties Questionnaire (SDQ)**   - Comprises 5 subscales: emotional symptoms; conduct problems; hyperactivity; peer problems; and pro-social behaviour. | **Strengths and Difficulties Questionnaire (SDQ)**   - Some of the strengths-based items on the hyperactivity scale, peer problems scale and prosocial scale reflect resilience constructs of empathy, self-reflection, goal orientation, and peer connection. | The study reported a Cronbach’s *a* = .85 indicating good internal consistency. The value of Cronbach’s alpha for four of the factors (emotional symptoms α = 0.70, conduct problems α = 0.78, hyperactivity α = 0.79, and prosocial α = 0.78) was acceptable, but the value for the peer problems domain was very low (α = 0.47). The peer relationships subscale is not a good fit for urban Aboriginal children; however, removing it does not significantly impact the fit of the model [24]. | *Content validity –* In a qualitative study by the same authors Aboriginal people in the Sydney region reported that the SDQ was acceptable overall and covered many important aspects of Aboriginal child and adolescent mental health [25]. However, some problems with the scale were noted, including the poor fit of the peer relationships subscale with Aboriginal concepts of appropriate social behaviour and relationships for children, as well as other limitations [24].  *Confirmatory Factor Analysis* was conducted to analyse the construct validity. The model fit by the CFA converged and provided a proper solution. Four factor loadings, three of which were from the peer relationships subscale, were unacceptably low (less than 0.4). The overall construct validity of the SDQ in this sample was acceptable but not “good” [24].  *Convergent validity –* the convergent validity of SDQ was assessed by estimating its relationship with between parental report of problematic behaviours in the past 6 months. The convergent validity of the SDQ was good [24]. |
| **Strong Souls**  A measurement instrument of Indigenous adolescent Social and Emotional Wellbeing (SEWB) developed and validated for Indigenous Australian adolescents. The instrument was found to have acceptable reliability with the sample of *n*=345 urban Australian Aboriginal children and adolescents. The instrument has demonstrated content and construct validity with the target population. Also, face validity and cultural validity was demonstrated with a pilot test sample of *n*=43, and discriminant validity was demonstrated with a pilot test sample of *n=*24, urban Australian Aboriginal children and adolescents [26]. | **Strong Souls**   - Measures constructs of anxiety, depression, suicide risk and resilience. | **Strong Souls**   - Items in the resilience construct measured both personal assets (humor; meaning through personal interest/skills; help seeking) and environmental resources (friendship; role models; family support; supportive adult relationship). | The study reported a Cronbach’s *a* = .70 suggesting acceptable internal consistency of Strong Souls overall, and within each factor with all 25 items showing reliability coefficients of at least 0.7 [26]. | *Content validity –* The tool was developed through comprehensive review of the Indigenous and general mental health literature. Final item selection was determined in relation to their meaningfulness and appropriateness for the target group through a widespread consultation process with Indigenous people and Indigenous mental health experts. Young Aboriginal adolescents provided feedback on the cultural and face validity of the questions, with this feedback informing the final version of Strong Souls [26].  *Face & Cultural validity –* The Strong Souls instrument was pilot tested with a sample of *n=*43 Aboriginal students under 17 years to assess cultural and face validity. Students were asked to evaluate the tools by indicating if items made sense, or were inappropriate (providing comments as applicable). The instrument demonstrated good face validity  on most items  *Discriminant validity -* The instrument was pilot tested with a sample of n=24 Aboriginal students over the age of 17 to assess discriminative validity. Good discriminant validity was demonstrated.  *Convergent validity* - Convergent validity was demonstrated through positive, significant correlations between the need for follow up and factor and scale scores of the three mental health factors; as well as through low but significant negative correlations between the need for follow up and resilience.  *Construct validity –* Exploratory factor analysis was used to explore construct validity. Factor analysis produced a 25-item, four-factor model accounting for 34.5% of the variance. Factor structure was consistent with the epidemiological literature, identifying constructs of anxiety, resilience, depression and suicide risk [26]. |
| **The Personal Wellbeing Index-School Children (PWI-SC)**  A standard measurement instrument of subjective wellbeing validated with Indigenous Australian school-children. The instrument was found to have good reliability with the sample of *n*=519 Indigenous Australian adolescents. While convergent validity was demonstrated, content validity was not [27]. | **The Personal Wellbeing Index-School Children (PWI-SC)**   - A measure of subjective-wellbeing addressing satisfaction in domains of: standard of living; achieving in life; relationships; safety; community-connectedness; and future security. | **The Personal Wellbeing Index-School Children (PWI-SC)**   - The domains of relationships and community-connectedness reflect resilience constructs of environmental resources. | The study reported a Cronbach’s *a* = .83 demonstrating good reliability with the target population [27]. | *Convergent validity* – subjective wellbeing (SWB) as measured by the PWI-SC accounted for 48% of the variance in the single item, measure of general life happiness (GLH). GLH and SWB correlate at .68, indicating convergent validity [27].  Principal Axis Factor Analysis demonstrated sound factor structure consistent with that intended by the developers of the PWI-SC [27]. |
| **Umyuangcaryaraq: ‘‘Reflecting’’**  ***Reflective Processes (RP)***  A culturally appropriate and strengths-based measurement instrument assessing reflective processes involved in thinking other the potentially consequences of alcohol use and abuse [16]. The five best functioning items were assembled as an outcome measure from the 12-item youth Reflective Processes scale [28].  Adapted from the adult Yup’ik Protective Factors scale designed for Alaska Native (AN) adults [20]. The instrument appears to be valid for use with the target population of Alaska Native adolescents, however reliability was suboptimal [16][28]. | **Reflective Processes (RP)**   - Assesses reflective processes along three dimensions: impacts on individual (self-subscale), family (family subscale) and the Alaska Native way of life (way of life subscale). | **Reflective Processes (RP)**   - Measures internal asset of self-awareness and future oriented reflection specifically related to alcohol and other drug use. | The study reported a Cronbach’s *a* = .49 in study 1 and Cronbach’s *a* = .38 in study 2. Test–retest Reliability for Study 1 = .36, and study 2 = .23, demonstrating suboptimal reliability values [16]. | *Content validity –* Content validity was established through expert review, cultural expert consultation, pilot testing, and further review to ensure equivalence with core concepts/original measures and cultural appropriateness [16].  ***Convergent validity* was shown in a separate study on instrument adaptation with Alaska Native adolescents** *-* The RP scale correlated in the expected direction with the Awareness of Connectedness Scale (ACS), Communal Mastery Scale (CMS), and Reasons for Life Scale (RFLS), and significantly but at lower magnitude with the Alaska Native Cultural Identification Scale (ANCI), providing support for convergent validity [28].  ***Discriminant validity*** **was shown in a separate study on instrument adaptation with Alaska Native adolescents** *-* Lack of association between RPS and White American Cultural Identification subscale (WACI) scores provided evidence for discriminant validity [28]. |
| **Yuuyaraqegtaar: ‘‘A Way to Live a Very Good, Beautiful Life’’**  ***Reasons for Life (RL)***  The five best functioning items were assembled as an outcome measure using a Graded Response Model from the 14-item Reasons for Life measurement instrument [16]. Assesses reasons why a person would not want to end life when feeling suicidal, emphasizing cultural beliefs and experiences that make life more enjoyable, worthwhile and provide meaning [20]. The instrument was shown to have acceptable internal consistency and test-retest reliability in on study, and content validity was demonstrated with the sample of *n*=54 Yup’ik Alaska Native youth [16]. | **Reasons for Life (RL)**   - Assesses four dimensions tapping reasons for life that are associated with others’ assessment of me, cultural and spiritual beliefs, sense of efficacy, and Family Responsibility. | **Reasons for Life (RL)**   - A culturally appropriate and strengths-based measure related to suicide risk. Taps into resources of Indigenous identity, cultural connectedness and community/family support/connection. | The study reported a Cronbach’s *a* = .78 in study 1 and Cronbach’s *a* = .69 in study 2. Test–retest Reliability for Study 1 = .71, and study 2 = .65, demonstrating good reliability [16].  In a **separate study with Alaska Native youth**, good internal consistency was demonstrated for all subscales (others’ assessment .76; Beliefs .71; Efficacy .70; Family responsibility .77) [20]. | *Content validity –* Content validity was established through expert review, cultural expert consultation, pilot testing, and further review to ensure equivalence with core concepts/original measures and cultural appropriateness [16]. |

**References**

1. Pritzker, S. and A. Minter, *Measuring adolescent resilience: An examination of the cross-ethnic validity of the RS-14.* Children and Youth Services Review, 2014. **44**: p. 328-333.

2. Ritchie, S.D., et al., *Promoting resilience and wellbeing through an outdoor intervention designed for Aboriginal adolescents.* Rural and Remote Health 2014. **14**(2523): p. 1-19.

3. Winterowd, C., et al., *Development of the American Indian Enculturation Scale to assist counseling practice.* American Indian and Alaska Native Mental Health Research, 2008. **15**(2): p. 1.

4. Stumblingbear-Riddle, G. and J.S.C. Romans, *Resilience among urban American Indian adolescents: Exploration into the role of culture, self-esteem, subjective well-being, and social support.* American Indian and Alaska native mental health research (Online), 2012. **19**(2): p. 1-19.

5. Bar-On, R., *The Bar-On model of emotional-social intelligence (ESI).* Psicothema, 2006. **18 Suppl**(1): p. 13-25.

6. Parker, J.D.A., et al., *Generalizability of the emotional intelligence construct: A cross-cultural study of North American aboriginal youth.* Personality and Individual Differences, 2005. **39**(1): p. 215-227.

7. DeJong, J.A. and J.M. Hektner, *L3 therapuetic model site* American Indian and Alaska Native Mental Health Research (Online), 2006. **13**(2): p. 79-122.

8. Hall, P.S. and J.A. DeJong, *Level 1 therapuetic model site.* American Indian and Alaska Native Mental Health Research (Online), 2006. **13**(2): p. 17-51.

9. Spears, B., et al., *Level 2 therapeutic model site.* American Indian and Alaska Native Mental Health Research (Online), 2006. **13**(2): p. 52-78.

10. Hanson, T.L. and J.O. Kim, *Measuring Resilience and Youth Development: The Psychometric Properties of the Healthy Kids Survey*, in *Issues & Answers Report, REL 2007–No. 034*. 2007, Department of Education, Institute of Education Sciences, National Center for Education Evaluation and Regional Assistance, Regional Educational Laboratory West: Washington, DC: U.S.

11. Dobia, B., et al., *Aboriginal Girls Circle: Enhancing Connectedness and Promoting Resilience for Aboriginal Girls: Final Pilot Report*. 2014, University of Western Sydney: Sydney.

12. Lowe, J., *Cherokee Self-Reliance.* Journal of Transcultural Nursing, 2002. **13**(4): p. 287-295.

13. Lowe, J., et al., *Community partnership to affect substance abuse among Native American adolescents.* The American Journal of Drug and Alcohol Abuse, 2012. **38**(5): p. 450-455.

14. Snowshoe, A., et al., *Development of a cultural connectedness scale for First Nations youth.* Psychological Assessment, 2015. **27**(1): p. 249-259.

15. Fok, C.C.T., et al., *Multicultural Mastery Scale for youth: Multidimensional assessment of culturally mediated coping strategies.* Psychological Assessment, 2012. **24**(2): p. 313.

16. Mohatt, G.V., et al., *Feasibility of a community intervention for the prevention of suicide and alcohol abuse with Yup'ik Alaska Native youth: The Elluam Tungiinun and Yupiucimta Asvairtuumallerkaa studies.* American Journal of Community Psychology, 2014. **54**(1-2): p. 180-186.

17. Fok, C.C.T., et al., *The Brief Family Relationship Scale: A brief measure of the relationship dimension infamily functioning.* Assessment, 2014. **21**(1): p. 67-72.

18. Diener, E., et al., *New well-being measures: Short scales to assess flourishing and positive and negative feelings.* Social Indicators Research, 2010. **97**(2): p. 143-156.

19. Haswell, M.R., et al., *Psychometric validation of the Growth and Empowerment Measure (GEM) applied with Indigenous Australians.* Australian and New Zealand Journal of Psychiatry, 2010. **44**(9): p. 791-799.

20. Allen, J., et al., *A Protective Factors Model for Alcohol Abuse and Suicide Prevention Among Alaska Native Youth.* American Journal of Community Psychology, 2014. **54**(1): p. 125-139.

21. Karcher, M.J. and D. Sass, *A multicultural assessment of adolescent connectedness: Testing measurement invariance across gender and ethnicity.* Journal of Counseling Psychology, 2010. **57**(3): p. 274-289.

22. Procidano, M.E. and K. Heller, *Measures of perceived social support from friends and from family: three validation studies.* American journal of community psychology, 1983. **11**(1): p. 1.

23. Marsh, H.W., et al., *A Short Version of the Self Description Questionnaire II: Operationalizing Criteria for Short-Form Evaluation With New Applications of Confirmatory Factor Analyses.* Psychological Assessment, 2005. **17**(1): p. 81-102.

24. Williamson, A., et al., *The construct validity of the strengths and difficulties questionnaire for Aboriginal children living in urban New South Wales, Australia.* Australian Psychologist, 2014. **49**(3): p. 163-170.

25. Williamson, A., et al., *Acceptability of an emotional and behavioural screening tool for children in Aboriginal Community Controlled Health Services in urban NSW.* Australian and New Zealand Journal of Psychiatry, 2010. **44**(10): p. 894-900.

26. Thomas, A., et al., *Strong Souls: Development and validation of a culturally appropriate tool for assessment of social and emotional well-being in Indigenous youth.* Australian and New Zealand Journal of Psychiatry, 2010. **44**(1): p. 40-48.

27. Tomyn, A.J., J.M. Norrish, and R.A. Cummins, *The subjective wellbeing of Indigenous Australian adolescents: Validating the Personal Wellbeing Index-School Children.* Social Indicators Research, 2013. **110**(3): p. 1013-1031.

28. Allen, J., et al., *Umyuangcaryaraq "Reflecting": Multidimensional assessment of reflective processes on the consequences of alcohol use among rural Yup'ik Alaska Native youth.* The American Journal of Drug and Alcohol Abuse, 2012. **38**(5): p. 468-475.
